# Autologous bone-marrow mononuclear stem cell therapy in patients with stroke: a meta-analysis of comparative studies

**DOI:** 10.1186/s12938-020-00819-7

**Published:** 2020-09-29

**Authors:** Sorayouth Chumnanvej, Siriluk Chumnanvej

**Affiliations:** 1 Neurosurgery Division, Department of Surgery, Faculty of Medicine Ramathibodi Hospital, Mahidol University, Bangkok, Thailand; 2 Department of Anesthesiology and Operating Room, Phramongkutklao Hospital, Bangkok, Thailand

**Keywords:** Stem cells, Mesenchymal stem cell transplantation, Stroke, Bone marrow, Random allocation

## Abstract

**Background:**

There is a need to promote recovery after stroke with novel therapeutic interventions. Of them, bone-marrow mononuclear cell (BM-MNC) therapy offers promising outcomes in preclinical and clinical models.

**Aims:**

To investigate the efficacy and safety of BM-MNCs versus traditional medical care of stroke patients.

**Summary of review:**

A meta-analysis was conducted involving controlled prospective studies and randomized clinical trials (RCTs) which investigated the changes in the scores of neurological functions (the National Institutes of Health Stroke Scale [NIHSS]), the indices of functional recovery (the Barthel Index [BI] and the modified Rankin scale [mRS]) at 3 and 6 month post-transplantation. A total of nine studies (five RCTs) recruited 469 stroke patients (65.5% males, 49.25% received the intervention). There were no significant differences in NIHSS, BI, or mRS scores after 3 months of follow-up. However, the BI indices of BM-MNCs-receiving patients improved significantly after 6 months (standardized mean difference = 1.17, 95% confidence interval, 0.23 to 2.10, *P* = 0.01) as compared to traditional treatment. The risk of mortality and adverse events and the proportion of patients with favorable outcomes (mRS ≤ 3) were similar in both groups.

**Conclusion:**

Both the BM-MNCs and medical stroke treatment have similar outcomes in terms of safety and short-term efficacy, while the effect of therapy is significant only after 6 months. More well-designed, large sized RCTs are needed to confirm the efficacy of stem cell therapy over long periods of follow-up.

## Background

Stroke is an acute life-threatening neurologic disorder which comprises of rupture or occlusion of brain blood vessels. Ischemic stroke incidents represent the majority of cases (87%), and the rest are hemorrhagic. Globally, the disease accounted for approximately 5.5 million deaths and 116.4 million disability-adjusted life-years in 2016, 80% of whom were reported in low- and middle-income countries [1]. Besides, about 795,000 individuals experience a new or recurrent stroke in the United States [2]. Furthermore, based on future projection analyses, there will be a 20% increase in stroke prevalence among American adults during the period between 2012 and 2030 [3]. This underscores the importance of controlling such a growing burden.

However, since post-stroke pathophysiological responses are perplexing, there is no currently specific therapy that mitigates the damage resulting from stroke. In particular, recombinant tissue plasminogen activator (r-tPA) remains the mainstay treatment for ischemic stroke incidents. Nevertheless, only 3–9% of patients receive r-tPA, because the treatment is eligible only within 3–4.5 h of symptom onset [4]. Endovascular mechanical thrombectomy has been recently introduced, showing improved functional outcomes in patients with severe stroke [5]. Nonetheless, the applicability of these approaches is either limited to distinct patient populations or still under development.

Therefore, there is a need to promote recovery in stroke patients via new therapeutic options. Early preclinical investigations on animal models have revealed promising outcomes of bone-marrow mononuclear cells (BM-MNCs) to reduce the infarct size and to enhance functional recovery in myocardium, limb, and cerebral ischemia models [6–10] These regimens were first identified in the bone-marrow stromal cells in the late 1960s [11]. MNCs entail several types of stem cells, including mesenchymal stem cells (MSCs) and hematopoietic stem cells (HSCs). MSCs can be isolated and amplified from bone marrow, have the ability to differentiate to neurons, and can be minimally rejected in allogenic transplantation.

In the clinical practice, clinical trials showed that BM-MNCs therapy is safe and feasible, although there is a considerable variation in study designs, the route of administration, and the time window of each trial. The outcomes of comparative investigations are usually integrated into those of cohort studies or single-arm clinical trials leading to misleading results. Therefore, in the present review, we sought to analyze the efficacy and safety of BM-MNC therapies in patients with different types of stroke, considering studies employing at least two cohorts of patients to contrast the impact of BM-MNCs with control subjects.

## Results

### Outcomes of the search process

Initially, a total of 202 records were obtained across all databases, of which 18 duplicates were removed. Additionally, 3 articles were identified from the bibliographies of screened articles. Therefore, 187 records were screened for eligibility. The full-article version was downloaded for 10 articles, where one article was excluded due to the lack of primary outcomes expressed as numerical variables [16]. Ultimately, nine studies were included in the quantitative analysis.

### Characteristics of the included studies

As shown in Table 1, studies were published between 2005 and 2019; six of which were conducted in Asian countries [17–22], while other studies were published in countries located in Africa [23], North America [24], and Europe [25] (one study in each). Five RCTs were included (55.56%), whereas the remaining studies employed two comparative arms without a randomization of patients. The intervention was given via intra-arterial (IA) injection in four studies [21, 23–25] through intravenous (IV) infusion in four studies [17–20] or it was directly injected to the perihemorrhage area in patients with hemorrhagic stroke [22].

**Table 1:** Characteristics of the included studies

Author	Country	Design	MOA	Cell dose	Stroke type	Day of the Procedure^a^	No. of patients		Gender (M/F)		Age		NOS
							*I*	*C*	*I*	*C*	*I*	*C*	
Bang et al. [17]	South Korea	RCT	IV	50 × 10^6^ twice	IS	7	5	25	4/1	14/11	63.0 ± 7.5	59.3 ± 11.5	NA
Lee et al. [18]	South Korea	RCT	IV	5 × 10^7^	IS	7	16	36	8/8	26/10	64.9 ± 14.5	64.0 ± 11.6	NA
Moniche et al. [25]	Spain	Non-RCT	IA	NA	IS	5–9	10	10	5/5	7/3	66.9 ± 13.9	67.4 ± 12.7	7
Bhasin et al. [19]	India	Non-RCT	IV	50-60 × 10^6^	IS & ICH	23	20	20	18/2	17/3	45.1 ± 12.1	45.2 ± 11.8	6
Li et al. [22]	China	Non-RCT	IC	9.47 × 10^5^	ICH	5–7	60	40	37/23	23/17	56.3 ± 2.9	55.9 ± 4.7	6
Prasad et al. [20]	India	RCT	IV	268 × 10^6^	Subacute IS	NA	60	60	41/19	36/24	50.7 ± 11.6	52.5 ± 12.1	NA
Ghali et al. [23]	Egypt	Non-RCT	IA	1 × 10^6^	Subacute IS	22	21	18	12/9	10/8	55.5 ± 5.3	56.25 ± 4.5	6
Bhatia et al. [21]	India	RCT	IA	6.1 × 10^8^	Subacute IS	7	10	10	6/4	8/2	57 ± 12.2	66.0 ± 7.3	NA
Savitz et al. [24]	USA	RCT	IA	3.08 × 10^6^	IS	11–17	29	19	20/9	15/4	59.3 ± 10.03	62.9 ± 10.81	NA

*C* control, *F* female, *I* intervention, *IA* intra-arterial, *IC* intracranial (perilesional), *ICH* Intracerebral hemorrhage, *IS* ischemic stroke, *IV* intravenous, *M* male, *MOA* mode of administration, *NOS* Newcastle-Ottawa Scale score, *RCT* randomized clinical trial

^a^Indicates the number of days after stroke to perform bone marrow extraction

In general, 469 (65.46% males) patients were recruited in all studies. Of them, 231 (49.25%) were allocated to the intervention group, for whom BM-MNCs and MSC therapies were given to 183 and 48 patients, respectively. Regarding stroke type, 103 patients (21.96%) had experienced a hemorrhagic stroke, while 187 (39.87%) and 179 (38.17%) patients had had acute and subacute ischemic stroke before transplantation, respectively.

### Quality assessment and risk of bias

**Fig. 1:**
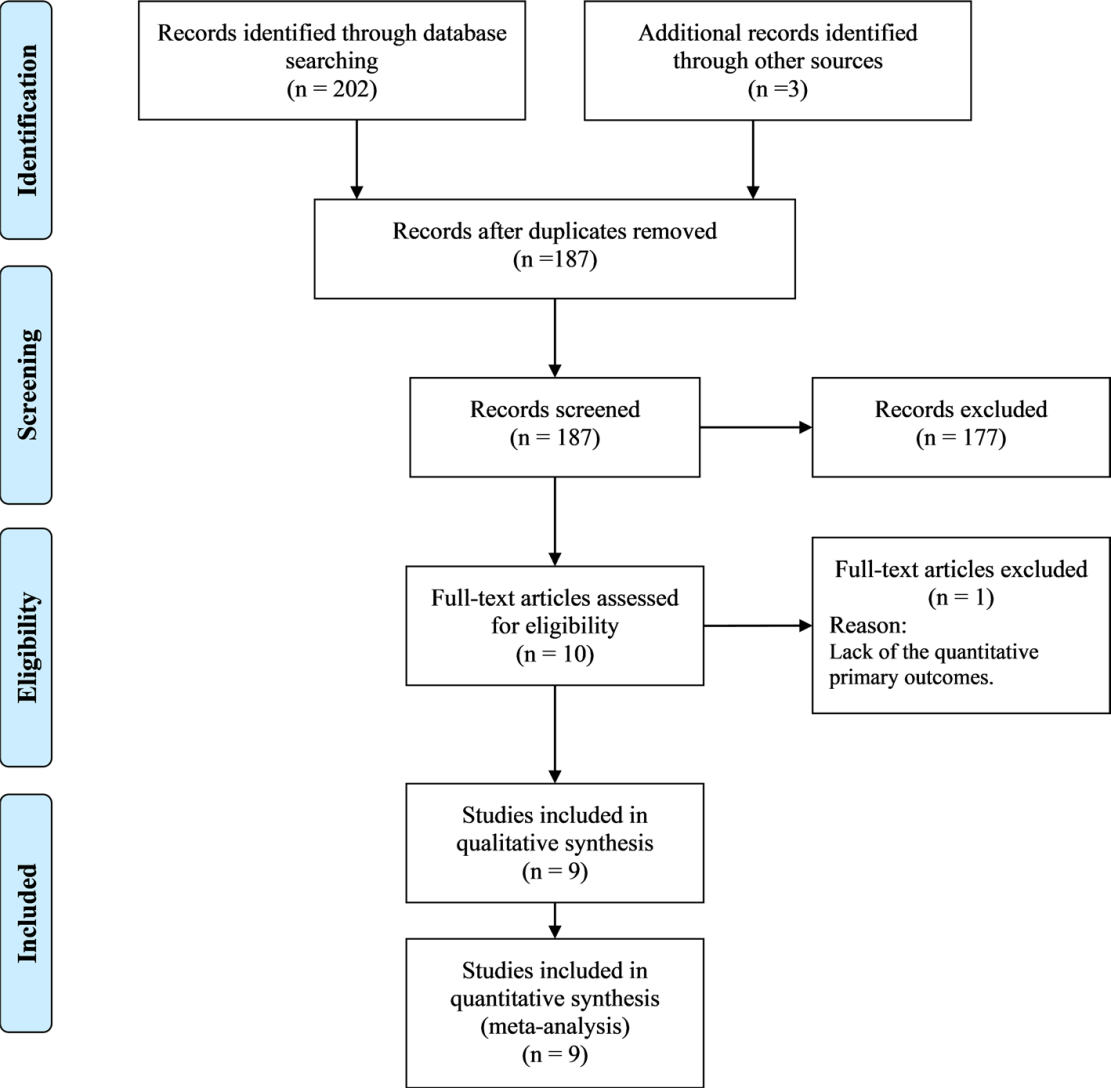
Summary of the risk of bias for each included randomized clinical trial

As shown in Fig. 1, risk of bias assessment of RCTs indicated that the recruited patients were randomized using a randomization table [17, 18, 21], permuted block randomization [20], or a specific computer software (Fig. 1) [24]. Of note, although strict measures were undertaken to ensure adequate blinding of the observers/clinicians during allocation, subsequent experimental procedures were not blinded in four (out of five) studies [17, 18, 20, 21] because of the obvious transplantation procedures as compared to medical treatment. However, Savitz et al. [24] used a sham-controlled group and hence they adequately blinded all personnel/patients. Regarding non-controlled studies, the NOS score was ≥ 6 for all studies, indicating a high-methodological quality (Table 1).

### Primary outcomes

**Fig. 2:**
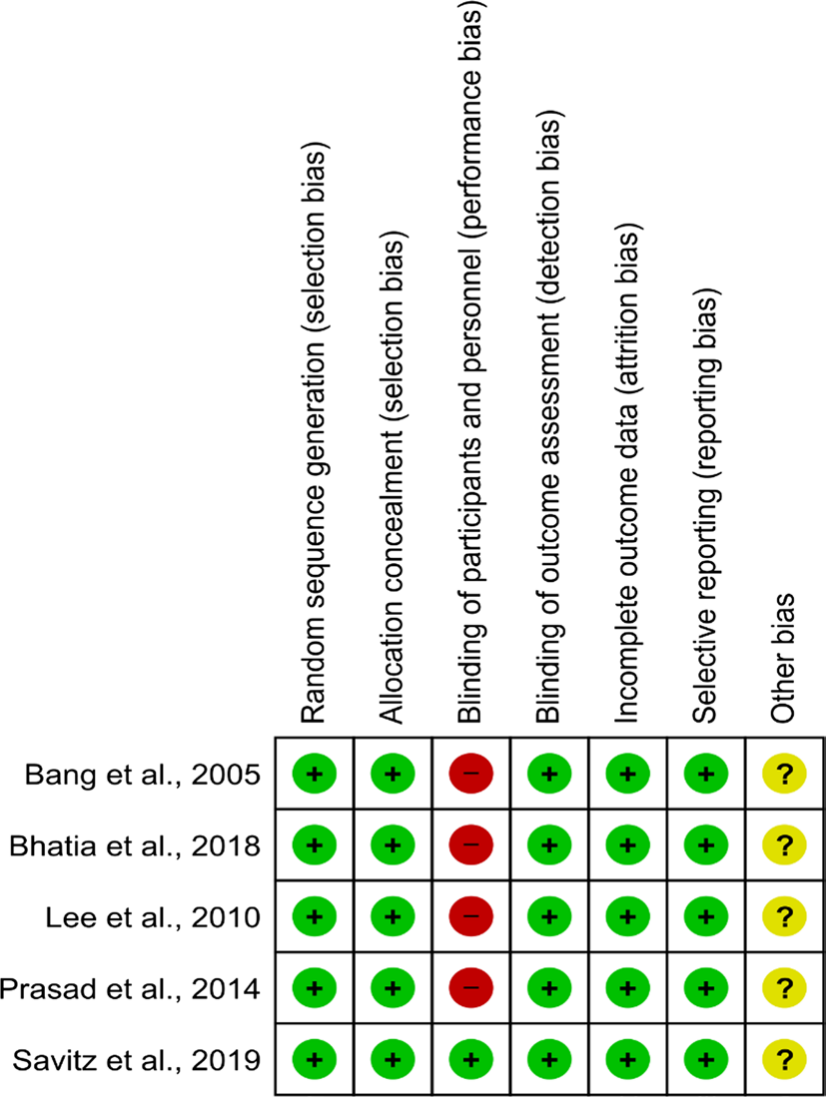
Forest plot showing the standardized mean difference in NIHSS scores at follow-up among patients with stroke. df: degree of freedom; *T*^2^: Tau-squared test (indicating between-study variance)

The quantitative primary outcomes for the NIHSS as an index of neurological deficit were available in six studies [17, 20, 22–25]. The scores were consistently lower in both groups at follow-up as compared to baseline values. However, there was no statistically significant difference between the BM-MNC-receiving groups and the control groups at 3 months (SMD = 0.08, 95% CI − 0.97 to 1.14, *P* = 0.87) and 6 months (SMD = − 0.71, 95% CI − 2.39 to 0.97, *P* = 0.41) after transplantation. Notably, there was a significant heterogeneity between studies (*I*^2^ = 95%, *P* < 0.001, Fig. 2).

**Fig. 3:**
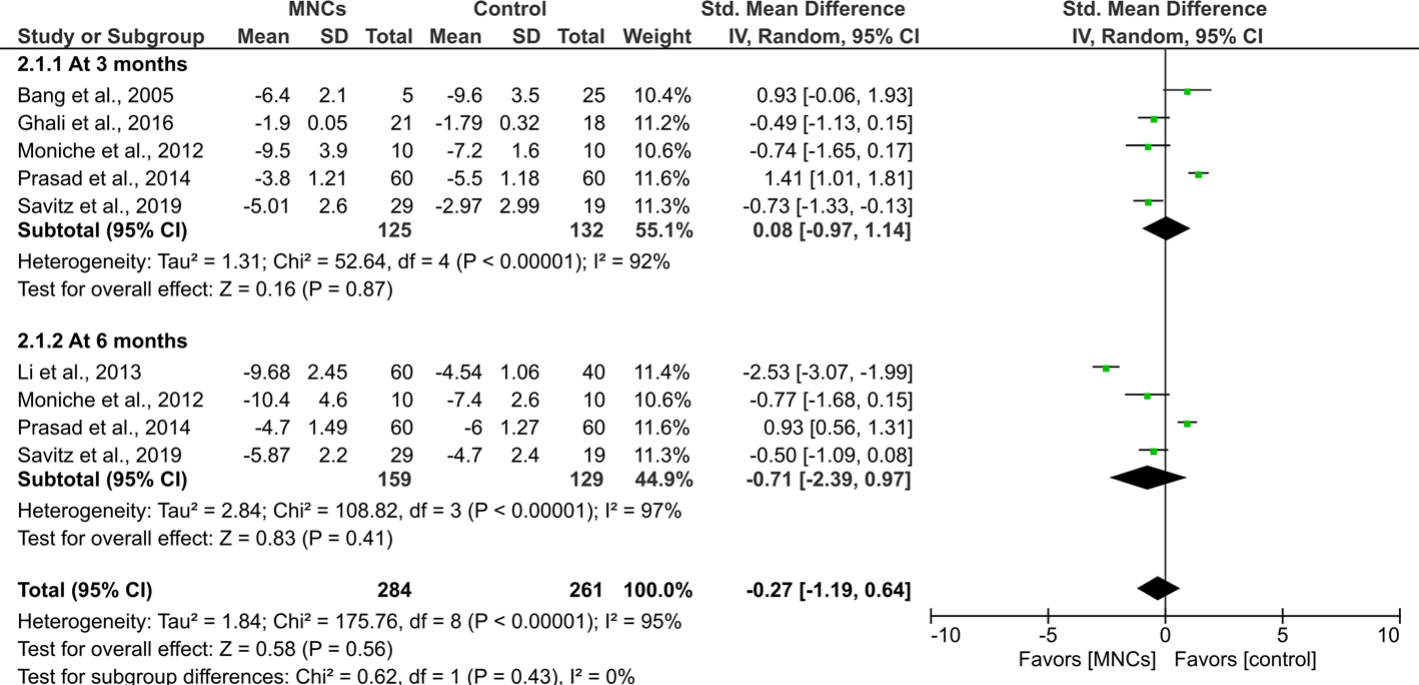
Forest plot showing the standardized mean difference in BI scores at follow-up among patients with stroke. df: degree of freedom; *T*^2^: Tau-squared test (indicating between-study variance)

**Fig. 4:**
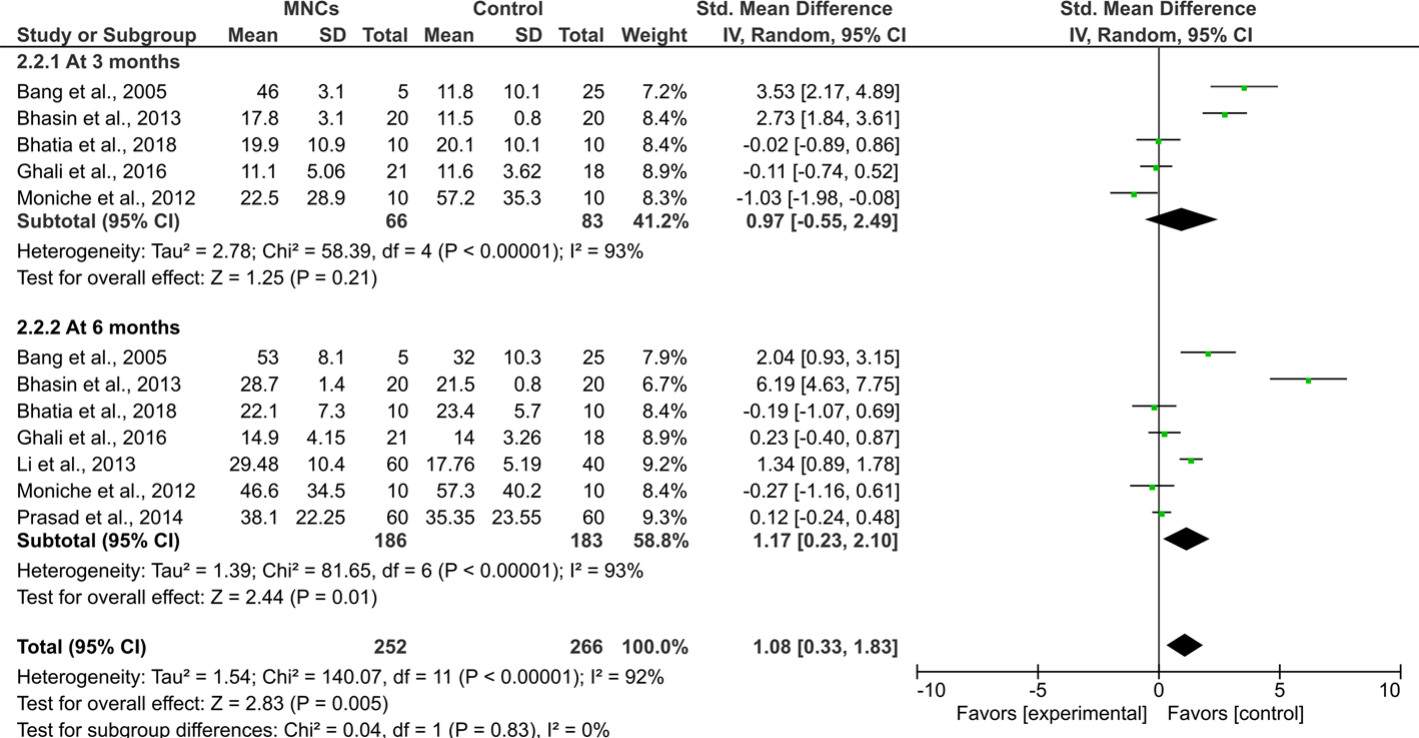
Forest plot showing the odds ratios of stroke patients with favorable outcomes who underwent BM-MNCs transplantation as compared to control groups

Regarding the values of the BI index, which were investigated in seven studies [17, 19, 23, 25], the difference between the intervention and control groups was only significant at 6 months after the procedure (SMD = 1.17, 95% CI 0.23 to 2.10, *P* = 0.01) with a significant heterogeneity between studies (*I*^2^ = 93%, *P* < 0.001, Fig. 3). Nevertheless, the proportions of patients who had favorable outcomes following the transplantation procedures were not significantly different than those allocated to the control groups (OR = 1.44, 95% CI 0.81 to 2.56, *P* = 0.22) and the studies were homogenous (*I*^2^ = 12%, *P* = 0.33, Fig. 4).

Subgroup analysis was performed on the primary outcomes with significant heterogeneity when at least five studies were included in the analysis. This was based on the route of administration (IA, IV, or intracrianial) and cell types (BM-MNCs or MSC). The analysis revealed that the studies became homogenous when they were grouped based on the route of administration. More specifically, IA injection of BM-MNCs significantly improved NIHSS scores (SMD = − 0.64, 95% CI − 1.03 to − 0.25, *P* = 0.001) as compared to traditional medical treatment at 3 months of follow-up (Table 2).

**Table 2:** Subgroups analysis of the primary outcomes that showed in-between-study heterogeneity

Item	Parameter	Studies	Patients	SMD	*P*	*I*^2^ % (model)
Mode of administration						
NIHSS (3 months)	IA	3	107	− 0.64 [− 1.03, − 0.25]	0.001^a^	0 (*F*)
	IV	2	150	1.35 [0.97, 1.72]	< 0.001^a^	0 (*F*)
BI (3 months)	IA	3	79	− 0.33 [− 0.90, 0.24]	0.26	34 (*F*)
	IV	2	70	2.97 [2.23, 3.71]	< 0.001^a^	0 (*F*)
BI (6 months)	IA	4	119	1.37 [− 0.61, 3.34]	0.18	95 (*R*)
	IV	2	150	1.01 [− 0.87, 2.88]	0.29	90 (*R*)
	IC	1	100	1.34 [0.89, 1.78]	NA	NA
Cell type						
NIHSS (3 months)	BM-MNCs	3	188	0.57 [0.26, 0.89]	< 0.001^a^	95 (R)
	MSC	2	69	− 0.07 [− 0.61, 0.47]	0.79	82 (*R*)
BI (3 months)	BM-MNCs	3	80	0.56 [− 1.63, 2.76]	0.61	94 (*R*)
	MSC	2	69	1.66 [− 1.91, 5.23]	0.36	96 (*R*)
BI (6 months)	BM-MNCs	5	300	1.24 [0.01, 2.48]	0.05	95 (*R*)
	MSC	2	69	1.08 [− 0.69, 2.84]	0.23	87 (*R*)

*BI* the Barthel Index, *BM*-*MNCs* bone marrow mononuclear cells*, F* a fixed-effects model, *IA* intra-arterial, *IC* intracranial (perilesional), *IV* intravenous, *MSCs* mesenchymal stem cells, *NIHSS* the National Institutes of Health Stroke Scale, *R* random-effects model, *SMD* standardized mean difference

^a^Indicates statistically significant differences

### Secondary outcomes

Patients who had undergone BM-MNCs procedures and control groups had no significant differences in the risk of all reported side effects, including partial seizures, low-grade fever, infection, recurrent vascular episodes, and the incidence of tumor. Moreover, the risk of death was similar between both arms without a significant heterogeneity between studies (Table 3).

**Table 3:** Risk ratios of adverse events and mortalities among stroke patients

Adverse event	No. of studies	No. of patients	Model (H %)	RR [95% CI]	*P*
Partial seizures	3	120	F (0)	2.05 [0.74, 5.73]	0.17
Fever	3	240	F (0)	1.57 [0.69, 3.58]	0.29
Infection	4	218	F (12)	1.56 [0.78, 3.12]	0.21
Recurrent vascular episodes	4	259	F (27)	2.11 [0.73, 6.13]	0.17
Malignancy	2	148	F (33)	0.45 [0.08, 2.60]	0.37
Death	3	192	F (47)	0.81 [0.47, 1.38]	0.44

*CI* confidence interval, *F* a fixed-effects model, *H* heterogeneity, *RR* risk ratio

## Discussion

Notwithstanding the recent advancement in rehabilitative and therapeutic approaches, the burden of stroke remains devastating both on the healthcare and financial levels. Only a small proportion of patients could benefit from instant therapeutic interventions, accounting for 10% of total patients treated at specialized stroke centers [26]. Therefore, researchers strive to develop definite pharmacologic and biologic therapies to reverse impairment in stroke patients. In the present study, we investigated the efficacy and safety of BM-MNC therapy in stroke patients as compared to regular medical treatments. Based on the functional assessment of patients, the effect of BM-MNCs was only significant at 6 months of follow-up as revealed by the BI scores. When compared to patients receiving a traditional treatment, patients receiving BM-MNCs did not achieve significant differences in other functional scores and the potential adverse events following the onset of stroke.

However, some studies indicated favorable effects of BM-MNCs at follow-up, considering baseline measures as a reference parameter. Particularly, patients with subacute stroke had significant improvements in all the scores, including NIHSS, mRS, and BI, in the intervention group at 6 months [21, 23]. Similar trends were observed in hemorrhagic stroke [22]. Additionally, the therapeutic effects were apparent in other observational studies which employed single groups of treated patients [27, 28]. Presumably, BM-MNCs act by upregulating endogenous recovery mechanisms both local and distant locations from the infarct. The peripheral effects of MNCs may be attributed to immunomodulation and reducing post-stroke inflammation. In vivo and in vitro studies have indicated that BM-MNCs can inhibit the Nuclear factor-κB (NF-κB) and tumor necrosis factor-α, and they reduce microglial activation and astrogliosis [29, 30]. The central effects include increasing the release of angiogenic growth factors, neurotrophic factors, as well as enhancing the survival of neuroblasts, decreasing necrosis, and promoting neurogenesis [31–33] Therefore, the clinical outcomes of these therapies should be considered.

Indeed, these beneficial effects may partly explain the significant impact of MNCs on BI scores 6 months after transplantation. This indicates significant improvements in the functional recovery rather than the diminution of neurological deficits. Besides, Bang et al. [17] revealed that BM-MNCs-treated patients had less prominent cerebral atrophy than the control group as indicated by magnetic resonance imaging scans. This might support the ability of stem cells to exert a diffuse action throughout the brain. In addition, Mendonca et al. [34] demonstrated early effects after 7 days of the procedure as shown by a significant improvement in the hypo-perfusion by single-photon Emission computed tomography (SPECT) scan. However, the outcomes of this study were based on the findings of a single patient and the BM-MNC therapy was administered on the third day of admission. Seemingly, the improvements in such a case may not be ascribed to the effects of stem cells, since they may require longer times to differentiate to vascular endothelial cells or neurons.

On the other hand, the lack of a significant difference than usual medical treatment necessitates additional investigations. We appreciate the existence of control groups to minimize the effect of confounding variables, which could be further reduced by adequate randomization and implementing efficient study designs. By contrasting intervention and control groups, we were able to recognize the magnitude of the therapeutic benefits. Therefore, the benefits of stem cell therapy in stroke patients need to be further approved in large, well-designed RCTs.

Besides, the most efficacious route of administration should be appreciated. In the present study, we showed that IA injection has been associated with NIHSS improvements at 3 month post-transplantation. Endovascular infusion through the IA mode infuses stem cells directly into the blood vessel perfusing the affected tissue. This might be more advantageous, since the filtrating effect of peripheral organs would be bypassed. As a result, the effective dose delivered to the infarcted area will be increased with uniform distribution. However, there is an evidence in some preclinical and clinical investigations indicating no differences between IA and IV routes [20, 35]. Increasing the number of studies that use a specific route could possibly enhance our knowledge and support the benefits of stem cell therapy in the future.

The outcomes of the present meta-analysis may be limited with the inherent limitations of the used functional scores in RCTs and comparative studies. For example, while the NIHSS score has shown excellent inter- and intra-observer reliability as well as promising feasibility to assess patients, the validity of such a measure is moderate, particularly when it is considered as a measure of subsequent disability resulting from stroke [36]. Similarly, the BI index has moderate validity to correlate with infarct size, nursing time requirements, and the degrees of motor loss [37]. In patients with mild symptoms, the efficacy of the BI index seems to be more emphasized on basic activities of daily living. Finally, the mRS scale provides a brief assessment of functional recovery, but it offers broad functional parameters. Therefore, its efficacy is most suited for large RCTs [37].

Other limitations might have affected the results of the present meta-analysis. The included studies had small sample sizes, ranging between 20 and 120 patients. This would have contributed to the lack of significant differences between the intervention and control groups. Additionally, while subgroup analysis showed that the route of administration contributed to the statistical heterogeneity between studies, it is recommended to conduct further RCTs based on the efficacy and safety of IA injection, which yielded promising outcomes. Subsequently, concluding the outcomes in future meta-analyses may confirm the best way by which BM-MNCs could be given to stroke patients. Of additional note, most of the included RCTs failed to blind the participants and the observers due to the obvious differences between BM-MNCs infusion and the traditional medical treatment. Recruiting a sham-controlled group would overcome this limitation in other trials in the future.

## Conclusion

In conclusion, autologous BM-MNCs provided significant effects only on one indicator of functional recovery 6 month post-transplantation. It adds no additional benefits and causes no additional harms to stroke patients as compared to the traditional medical treatment. However, stem cell therapies given via the IA route seem to provide promising effects, which should be further investigated. Conducting future RCTs with large sample sizes, sham-controlled designs, and adequate blinding is warranted.

## Methods

A meta-analysis was conducted based on the guidelines of the Preferred Reporting Items for Systematic Reviews and Meta-Analyses (PRISMA) [12].

### Eligibility criteria

**Fig. 5:**
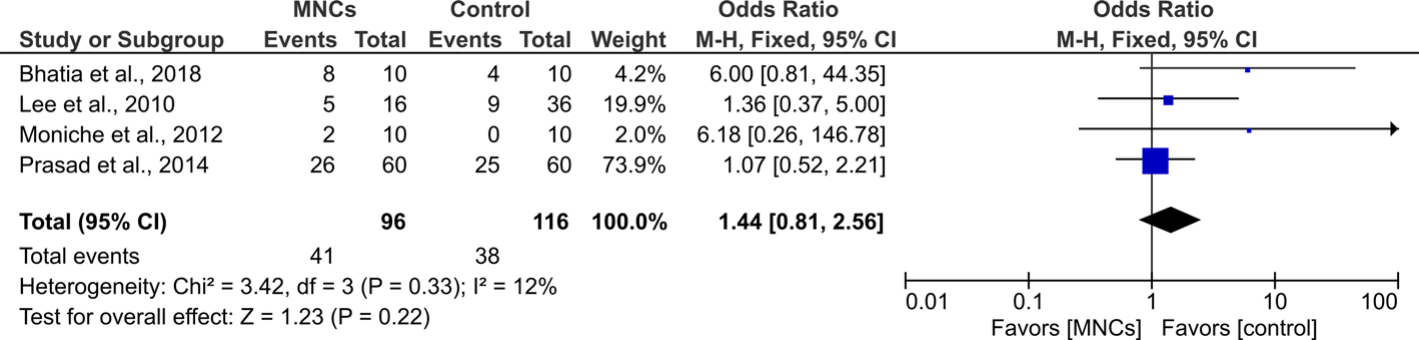
Flowchart showing the search process used in the present study

Eligible studies were randomized clinical trials (RCTs) as well as prospective and retrospective cohort studies which recruited two cohorts of stroke patients; an experimental group receiving BM-MNCs and a control group receiving a traditional stroke medical therapy. Adult patients (≥ 18 years) with an ischemic or hemorrhagic stroke in the acute to chronic stage were eligible. Studies reporting at least one clinical score indicating functional or neurological recovery/improvement were included. Non-randomized single-arm clinical trials, narrative reviews, case reports, and conference proceedings, as well as prospective and retrospective cohort studies recruiting a single intervention group of patients were excluded. Besides, articles written in a non-English language were not considered. As shown in Fig. 5, the number of included and excluded articles at the different phases was presented in a PRISMA flowchart.

### Primary and secondary outcomes

The primary outcomes included the difference in the measurements of neurological functions, which is indicated by the National Institutes of Health Stroke Scale (NIHSS) score, as well as the changes in the indices of functional recovery (the Barthel Index [BI]). Besides, primary outcomes included the number of patients with favorable outcomes in each study group, as indicated by a modified Rankin scale (mRS) of ≤ 3 at 6 month post-transplantation. Safety outcomes following transplantation procedures, including death, infection, tumor formation, etc., were considered secondary outcomes. Additionally, the reported recurrent vascular episodes, including stroke recurrence or coronary heart disease, were additional secondary outcome variables.

### Search strategy

The following scientific databases were searched for eligible studies for the objective of this meta-analysis: Embase, Cochrane Library, PubMed, and Google Scholar. The last access to such databases was on October 23, 2019. The search strategy was formulated using a specific combination of keywords and Boolean operators (i.e., AND and OR). The used search process in the PubMed database is demonstrated in Appendix 1.

### Study selection and data collection

The search process was conducted by two independent authors, who screened the titles and abstracts of all obtained records across databases. Subsequently, the results were uploaded to a reference manager (Endnote X7), where duplicate records were omitted. The bibliographies of screened articles were additionally searched for eligible studies. A spreadsheet was designated in Microsoft Excel for data extraction. The following data was collected for each individual study: (1) study-related data, including the date of publications, the last name of the first author, study design, and country; (2) intervention-related data, including the type of intervention (BM-MNC or MSC), route of administration, days after stroke to perform BM extraction, and cell dose; (3) patient-related data, including the mean (standard deviation[SD]) age of each study group, gender distribution, the number of patients and the type of stroke; (4) primary outcomes, including the reported means (SD) of NIHSS and BI obtained at baseline and at 3 and 6 months after transplantation as well as the frequency of patients with favorable neurological outcomes (mRS ≤ 3); (5) the frequency of patients with adverse events/death after transplantation. Any disagreement between authors was resolved by discussion.

### Risk of bias and the assessment of methodological quality

The RCTs were assessed using The Cochrane’s Risk of Bias Tool [13], including the assessment of random sequence generation, blinding, allocation concealment, incomplete outcome data, selective reporting, and other bias. Each study was assigned a judgement of “low risk”, “high risk”, or “unclear” independently by two authors. The results were depicted using the RevMan software (the Cochrane Collaboration, Oxford, United Kingdom). On the other hand, given that other comparative studies did not a randomized design, the Newcastle–Ottawa Scale (NOS) was used for the assessment of their methodological quality [14]. This includes an assessment of patient selection, comparability, and outcomes using a specific grading system, giving rise to a score ranging between 0 (lowest quality) and 8 (highest quality). Scores of high-quality and medium-quality studies were considered at ≥ 6 and ≥ 4, respectively.

### Statistical analysis

Statistical analysis was carried out using RevMan v 5.3 (the Cochrane Collaboration, Oxford, United Kingdom). Quantitative variables, including the scores of neurological functions and functional recovery, were expressed as mean ± SD. Median values and interquartile ranges were converted to means and SD as described previously [15]. Means and standard deviations were pooled and analyzed using standardized mean differences (SMDs) and their respective 95% confidence intervals (CIs). On the other hand, qualitative variables were extracted as frequencies and percentages. The numbers of patients with favorable outcomes were analyzed as odds ratios (ORs) and 95% CIs, while risk ratios (RRs) and 95% were used to estimate the risk of adverse events and mortality. Statistical heterogeneity between studies was assessed using a *I*^2^ test, where a random-effects model was applied at *I*^2^ ≥ 50%, and a fixed-effects model at lower values.

## Data Availability

All data generated or analyzed during this study are included in this published article.

## Appendix 1: The search process used in the PubMed database

#1 “Bone Marrow”[Title/Abstract]

#2 “Mononuclear”[Title/Abstract] OR “stem”[Title/Abstract] OR “mesenchymal”[Title/Abstract]

#3 “group*”[Title/Abstract] OR “compare*”[Title/Abstract] OR “control”[Title/Abstract] OR “random*”[Title/Abstract]

#4 “stroke”[Title/Abstract]

## Abbreviations

BI: The Barthel Index
BM-MNCs: Bone-marrow mononuclear cells
CIs: Confidence intervals
HSCs: Hematopoietic stem cells
IA: Intra-Arterial
IV: Intravenous
mRS: Modified Rankin Scale
MSCs: Mesenchymal stem cells
NF-κB: Nuclear Factor-κB
NIHSS: The National Institutes of Health Stroke Scale
NOS: Newcastle–Ottawa Scale
PRISMA: Preferred Reporting Items for Systematic Reviews and Meta-Analyses
RCTs: Randomized clinical trials
RRs: Risk ratios
r-tPA: Recombinant tissue plasminogen activator
SD: Standard deviation
SMDs: Standardized mean differences
SPECT: Single-photon emission computed tomography
